# A Meta-Analysis Evaluating the Effectiveness and Safety of Upadacitinib in Treating Rheumatoid Arthritis in Patients With Inadequate Response to Disease-Modifying Anti-Rheumatic Drugs

**DOI:** 10.7759/cureus.34384

**Published:** 2023-01-30

**Authors:** Viraj Panchal, Bhavya H Vyas, Barath Prashanth Sivasubramanian, Kanan Panchal, Harshank Patel

**Affiliations:** 1 Internal Medicine, Smt Nathiba Hargovandas Lakhmichand Municipal Medical College, Ahmedabad, IND; 2 Internal Medicine, Employees' State Insurance Corporation Medical College and Post Graduate Institute of Medical Science and Research, Chennai, IND; 3 Radiology, Sardar Vallabhbhai Patel Institute of Medical Sciences & Research, Ahmedabad, IND

**Keywords:** rheumatoid arthritis, meta-analysis, abt-494, upadacitinib, efficacy and safety

## Abstract

Upadacitinib, an oral *Janus kinase* (*JAK*) *inhibitor*, is used to manage rheumatoid arthritis. The objective was to generate statistical evidence from the existing data for *upadacitinib* efficacy and safety in various treatment regimens with different dosages in active rheumatoid arthritis patients. We searched PubMed, Cochrane, and ClinicalTrials.gov using PRISMA guidelines, providing data on the efficacy and safety of u*padacitinib* versus placebo in rheumatoid arthritis. 20% improvement in the American College of Rheumatology (ACR20) score response at 12 weeks was the primary outcome measure. Safety in adverse events, infections, or hepatic dysfunction was considered. The Mantel-Haenszel formula with random effect was used for the pooled odds ratio (OR) at a 95% confidence interval (CI) for dichotomous data. Meta-analysis was performed using RevMan version 5.4. Statistical heterogeneity was reported using I2 statistics; I2 > 75% was considered significant heterogeneity. A P value of less than 0.05 was considered significant. Data from 3233 patients were included in the analysis. The use of upadacitinib was associated with increased rates of achieving an ACR20 response compared with placebo (pooled OR 3.71; 95% CI 3.26-4.23; p-value <0.00001). Compared to a placebo, a 12 mg twice daily dose had the greatest effect, followed by a 15 mg once daily dose. Compared to the placebo, the incidence of any adverse event (pooled OR 1.66; 95% CI 1.36-2.02; p-value 0.0001) and infection (pooled OR 1.46; 95% CI 1.23-1.74; p-value 0.001) was found to be significantly higher in upadacitinib. Other adverse events, such as hepatic disorders and herpes zoster infections, were not statistically significant (p-value> 0.05). Maximum adverse events were seen at 12 mg twice daily. Upadacitinib, 15 mg once daily in combination with Methotrexate, was the most efficacious treatment regimen and was not associated with a significant risk for treatment-related adverse events in rheumatoid arthritis patients.

## Introduction and background

Rheumatoid arthritis is a chronic, systemic autoimmune disease characterized by persistent joint inflammation and extra-articular manifestations affecting many other organ systems. The etiology of rheumatoid arthritis is still unknown; however, the role of inflammation and various cytokines released with activation of CD4+ T-cells and the formation of antibodies against self-antigens such as rheumatoid factor (RF), anti-citrullinated protein antibodies (anti-CCP), and many more, that results in progressive joint destruction by the proliferation of synovial cells leading to synovitis and pannus formation, ultimately destroying the cartilage and ankylosis of the joints, is known [1,2]. Janus-activated kinases (JAKs), which are intracellular tyrosine kinases, act as a signaling mediators for multiple cytokines and growth factors involved in the pathogenesis of inflammation and autoimmune disorders [3]. Targeted synthetic drug therapy in the form of small-molecule JAK inhibitors is being developed clinically to treat rheumatoid arthritis. JAK inhibitors can modulate several inflammatory pathways involved in the pathogenesis of rheumatoid arthritis because of their ability to suppress the intracellular signaling of multiple cytokines [4,5].

Upadacitinib, an oral Janus kinase (JAK) inhibitor, is a more selective inhibitor of JAK 1 compared to JAK 2, JAK 3, and tyrosine kinase 2 (Tyk2). It has undergone multiple clinical trials in managing rheumatoid arthritis, either as a single agent or using Methotrexate (MTX) [6]. The efficacy and safety of upadacitinib are being investigated in patients who have had an inadequate response to conventional synthetic disease-modifying anti-rheumatic drugs (csDMARDs) like methotrexate and biologic disease-modifying anti-rheumatic drugs (bDMARDs), as well as in those patients who have had a sparse response to at least 1 anti-Tumor Necrosis Factor (TNF) agent [7-12].

However, due to a lack of adequate information regarding the efficacy and safety of upadacitinib in various treatment regimens with different dosages, we decided to do a meta-analysis of all published randomized controlled trials (RCTs) involving upadacitinib versus placebo in treating rheumatoid arthritis patients with poor responses to DMARDs.

## Review

Methods

Search Strategy

A meta-analysis was conducted according to the Preferred Reporting Items for Systematic Reviews and Meta-Analyses (PRISMA) guidelines [13]. We systematically searched PubMed, Cochrane Library, and ClinicalTrials.gov database for relevant clinical trials published after January 2000 until 2020. Search MeSH terms included the following: "upadacitinib," "ABT-494," "rheumatoid arthritis," "biologics," "Janus kinase inhibitors," and "randomized clinical trials." After removing duplicate and irrelevant studies, two investigators examined each potential article independently (VP, BV) to determine if it met the inclusion criteria. Any disagreement was settled through discussion.

Study Selection

All study related Randomized controlled trials (RCTs) using either: An adequate method of allocation concealment (e.g., sealed opaque envelopes), Studies that were double-blind, single-blind, or unblinded, Studies that included a comparison of Upadacitinib with placebo in patients with active rheumatoid arthritis were selected.

RCTs of Upadacitinib with placebo control trials in 18 years or older with active rheumatoid arthritis for more than 3 months in which patients had received csDMARDs: Methotrexate, sulfasalazine, or leflunomide for at least 3 months with a stable dose for at least 4 weeks before study entry and those studies that provided any available information on primary or secondary outcomes in terms of frequency, numbers or percentages were included for the analysis. Studies with upadacitinib RCTs with no placebo arm, outcome measures in a graphical presentation, unpublished research work or trials, observational studies, and preclinical studies were excluded.

Data were extracted from studies meeting the criteria. In some studies, data that the inquiry could not obtain was excluded. Data on the study design, the treatment comparator, in which a placebo was used, the dosage, the treatment response rate in terms of a 20% improvement in the ACR20 response at 12 weeks, and adverse events at the end of 12 weeks were collected.

Main Outcome Variables

Upadacitinib versus placebo in patients with rheumatoid arthritis who had an inadequate response to csDMARDs, bDMARDs, or anti-TNF agents. Primary efficacy outcome was measured as Treatment response rate (20% improvement in the ACR20 response at 12 weeks) [14]. Secondary safety endpoints included any adverse events, including an upper respiratory infection, nasopharyngitis, urinary tract infection, or worsening of rheumatoid arthritis. The development of any hepatic disorder, infection, severe infection, and herpes zoster infection was also noted.

Risk of Bias Assessment

The risk of bias for the included RCTs was assessed by two independent reviewers (V.P., BV) according to the guidelines in the Cochrane Handbook for Systematic Reviews of Interventions [15]. Different parameters such as selection bias, allocation bias, blinding, incomplete reporting of data, and any other type of bias were graded as low, unclear, or high risk.

Analysis

The Mantel-Haenszel formula with a random and fixed effect model was used to directly compare upadacitinib and placebo to calculate the pooled odds ratio (OR) at a 95% confidence interval (CI) for dichotomous data, and a forest plot was created. An OR of one means that the new treatment and the placebo have equivalent effects. If improvement is associated with higher scores on the outcome measure, an OR greater than one indicates the degree to which treatment is more efficacious than a placebo, and an OR less than one indicates the degree to which treatment is less efficacious than a placebo. Meta-analysis was performed using RevMan version 5.4. Statistical heterogeneity across studies was reported using I2 statistics. The I2 statistic of >75% was considered significant heterogeneity, and a p-value of <0.05 was considered significant.

Results

Baseline Characteristics 

**Figure 1:**
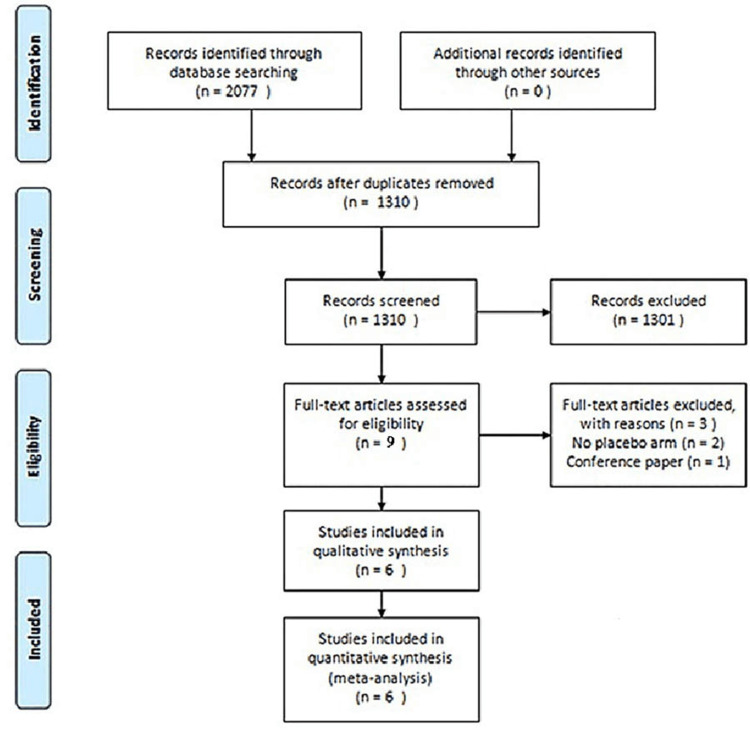
PRISMA flow diagram for search strategy.

A total of 9 studies were identified through an electronic or manual search and were selected for a full-text review based on the title and abstract details [6-12,16,17]. However, two of the nine were eventually excluded as they did not have a placebo arm [6,16], and one was excluded as it was a conference paper [17]. Ultimately, six RCTs involving 3233 patients met the inclusion criteria [7-12]. All the RCTs provided data related to both efficacy and safety events. As noted among the RCTs, we chose various dosages ranging from 3 to 30 mg of upadacitinib. The relevant features of the RCTs included in the meta-analysis are provided in Table 1 and Figure 1. Assessment of the risk of bias for included studies is shown in Table 2 of the appendices. 

**Table 1:** Baseline characteristics of included studies for meta-analysis. Source References: [7-12] RCT: Randomized controlled trials; csDMARDs: conventional synthetic disease-modifying anti-rheumatic drugs.

Study	Year	Design	Sample size	Intervention	Control
Kremer et al. [7]	2016	RCT phase 2b, multicentric worldwide	276	Upadacitinib of 3, 6, 12, or 18 mg twice daily + Methotrexate	Placebo matching twice daily + Methotrexate
Genovese et al. [8]	2016	RCT phase 2b, multicentric worldwide	299	Upadacitinib of 3, 6, 12, or 18 mg twice daily, 24 mg once daily + Methotrexate	Placebo matching twice daily or once daily + Methotrexate
Burmester et al. [9]	2018	RCT phase 3, multicentric worldwide	661	Upadacitinib 15 mg or 30 mg once daily + csDMARDs	Placebo once daily + csDMARDs
Genovese et al. [10]	2018	RCT phase 3, multicentric worldwide	498	Upadacitinib 15 mg or 30 mg once daily + csDMARDs	Placebo once daily + csDMARDs
Fleischmann et al. [11]	2019	RCT phase 3, multicentric worldwide	1302	Upadacitinib 15 mg once daily + Methotrexate	Placebo once daily + Methotrexate
Kameda et al. [12]	2020	RCT phase 2b/3, multicentric worldwide	197	Upadacitinib 7.5, 15, or 30mg once daily + csDMARDs	Placebo once daily + csDMARDs

Primary Efficacy Endpoints

**Figure 2:**
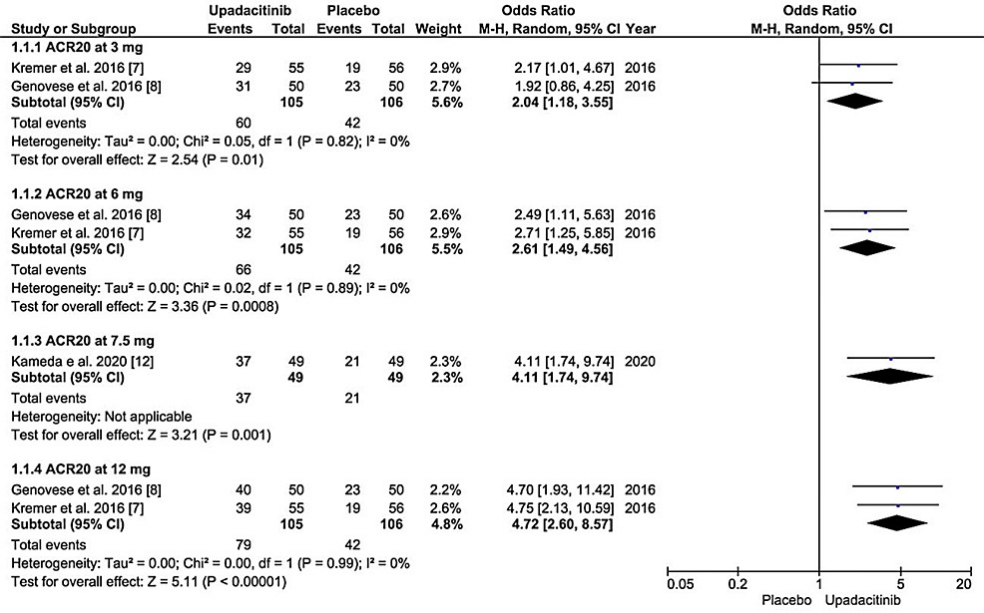
Forest plot showing the efficacy in improving ACR20 score at the end of 12 weeks among patients receiving upadacitinib (3, 6, 7.5, and 12 mg) compared to placebo. Source References: [7-12]

**Figure 3:**
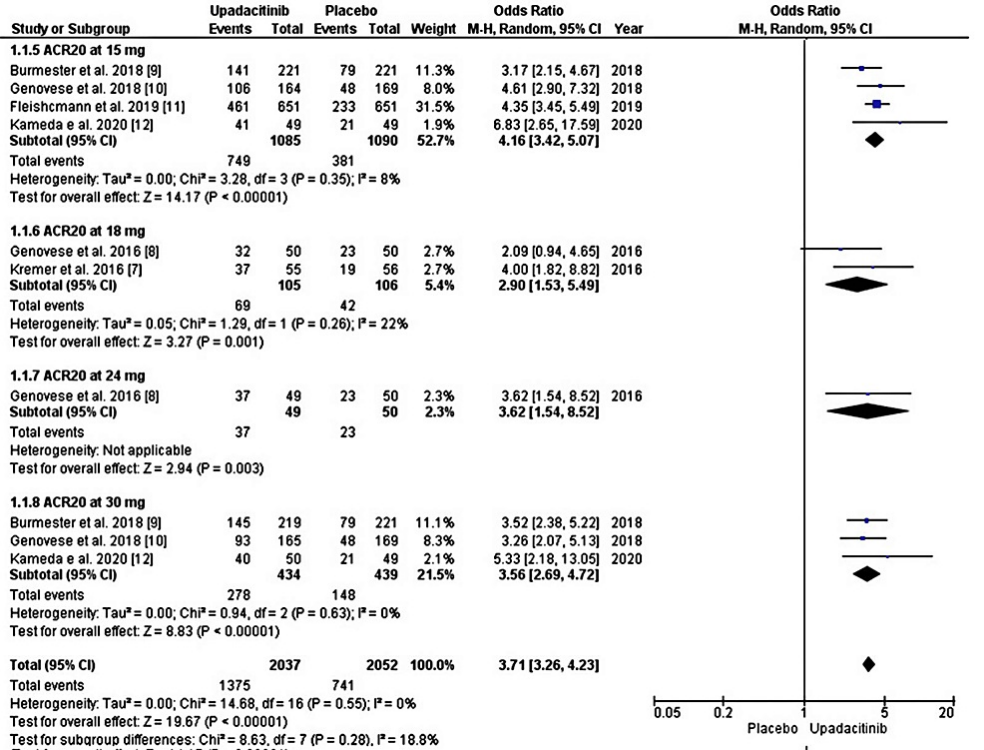
Forest plot showing the efficacy in improving ACR20 score at the end of 12 weeks among patients receiving upadacitinib (15, 18, 24, and 30 mg) compared to placebo. Source References: [7-12]

All six studies reported a 20% improvement in the ACR20 response at 12 weeks. In our population, 1375 out of 2037 patients (67.5%) achieved the ACR20 response in the upadacitinib group compared with 741 out of 2052 (36.1%) in the placebo group. The overall OR for all doses in the random effect was 3.71 and 95%. CI was 3.26 to 4.23, and the p-value was <0.00001, which was statistically significant, as shown in Figure 2 and Figure 3.

Overall dose heterogeneity was 0% with a P value of 0.55, which was insignificant, so sensitive analysis was not performed further. Compared to the placebo, 15 mg once daily upadacitinib had the highest OR of 4.16, 95% CI 3.42 to 5.07, and a p-value of 0.00001. All other doses of upadacitinib (3, 6, 7.5, 12, 18, 24, and 30 mg) were also statistically significant for achieving an ACR20 response at 12 weeks (p-value <0.05) compared to the placebo. 

Safety Outcomes

Five of the six included studies reported safety outcomes at the end of 12 weeks. As the study by Fleischmann R et al. [11] from the included studies showed the safety outcome measured at the end of 26 weeks, we had to exclude that study from analyzing the safety outcome. A total of 1254 different events were reported among 1386 patients receiving upadacitinib, while 1006 different types of events were seen among 1401 patients receiving a placebo.

At the subgroup level, 786 (56.7%) of 1386 patients who received upadacitinib reported any treatment-related adverse events, while 643 (45.7%) of 1401 patients who received placebo reported any treatment-related adverse events, resulting in a pooled OR of 1.66, 95% CI of 1.36 to 2.02, and a statistically significant p-value of 0.0001. Only doses of 3 mg twice daily, 7.5 mg and 15 mg once daily did not show a significant difference in any treatment-related adverse events when compared with placebo with a p-value of >0.05 (pooled OR 1.4, 95% CI of 0.8 to 2.46, p value=0.24 at 3 mg; pooled OR of 1.51, 95% CI of 0.68 to 3.36, p value=0.31 at 7.5 mg; pooled OR of 1.20, 95% CI of 0.92 to 1.57, p value=0.18 at 15 mg); and the maximum occurrence of any adverse event was seen at 12 mg twice daily, OR 3.10, 95% CI 1.76 to 5.49, p-value <0.0001.

When overall upadacitinib dosages were compared with placebo for the occurrence of infection, the pooled OR was 1.46, 95% CI 1.23 to 1.74, and the p-value was 0.0001, which was statistically significant. Individual doses of 12 mg twice daily and 15 and 30 mg once daily resulted in statistically significant infections with a p-value of <0.05 (pooled OR was 2.10, 95% CI of 1.10 to 3.99, p value=0.02 at 12 mg twice daily; pooled OR was 1.36, 95% CI of 1.01 to 1.83, p value=0.05 at 15 mg once daily; pooled OR of 1.58, 95% CI of 1.06 to 2.36, p value=0.03 at 30 mg once daily). Similarly, only 30 mg once daily dose of upadacitinib showed a significant occurrence of severe infection, with an OR of 5.25, 95% CI of 1.13 to 24.48, and a p-value of 0.03; at other dosages, it was not found to be statistically significant with a p-value of >0.05 (pooled OR was 0.33, 95% CI of 0.01 to 8.36, p value=0.94 at 3 mg; pooled OR of 0.33, 95% CI of 0.01 to 8.36, p value=0.5 at 6 mg; pooled OR was 1.01, 95% CI of 0.10 to 9.86, p value=0.99 at 12 mg; pooled OR was 1.96, 95% CI of 0.34 to 11.41, p value=0.45 at 15 mg; pooled OR was 0.33, 95% CI of 0.01 to 8.36, p value=0.5 at 18 mg). Occurrences of hepatic dysfunction or herpes zoster infection were not statistically significant, even at individual doses with a p-value of >0.05.

For hepatic dysfunction, pooled OR was 10.1, 95% CI of 0.10 to 9.86, p value=0.99 at 3 mg; pooled OR was 0.33, 95% CI of 0.01 to 8.36, p value=0.50 at 6 mg; pooled OR was 0.19, 95% CI of 0.01 to 4.10, p value=0.29 at 7.5 mg; pooled OR was 0.33, 95% CI of 0.01 to 8.36, p value=0.50 at 12 mg; pooled OR was 0.89, 95% CI of 0.34 to 2.34, p value=0.82 at 15 mg; pooled OR was 2.96, 95% CI of 0.44 to 19.86, p value=0.26 at 18 mg; pooled OR was 1.13, 95% CI of 0.45 to 2.86, p value=0.74 at 30 mg. Similarly, for herpes zoster infection, pooled OR was 0.96, 95% CI of 0.14 to 6.71, p value=0.97 at 3 mg; pooled OR was 0.19, 95% CI of 0.02 to 1.69, p value=0.14 at 6 mg; pooled OR was 1.00, 95% CI of 0.06 to 16.45, p value=1.00 at 7.5 mg; pooled OR was 0.50, 95% CI of 0.04 to 5.68, p value=0.58 at 12 mg; pooled OR was 0.75, 95% CI of 0.14 to 4.00, p value=0.73 at 15 mg; pooled OR was 0.50, 95% CI of 0.04 to 5.68, p value=0.58 at 18 mg; pooled OR was 5.32, 95% CI of 0.25 to 113.61, p value=0.28 at 24 mg; pooled OR was 3.02, 95% CI of 0.80 to 11.40, p value=0.10 at 30 mg. heterogeneity was <75% in safety outcomes with a p-value of >0.05, hence sensitive analysis was not performed. 

Discussion

It is well known that the Janus kinase pathway plays a significant role in the pathophysiology of inflammation and autoimmune disorders. The ability of a drug like upadacitinib, as a JAK inhibitor, to suppress the intracellular signals has the advantage of modulating several inflammatory pathways involved in the progression of rheumatoid arthritis. Upadacitinib, eventually, is responsible for decreasing inflammation [4]. 

Our study pooled 3233 patients from six multicenter, double-blinded, randomized clinical trials. It showed that the use of upadacitinib and csDMARDs is associated with an increased rate of achieving the primary efficacy endpoint: a 20% improvement in the ACR20 response at 12 weeks compared with a placebo. Maximum significant efficacy was found with the dosage of 15 mg once daily of upadacitinib (OR 4.16; 95% CI 3.42-5.07; p-value <0.00001) along with csDMARDs. Evaluation of the safety endpoints demonstrated a significant difference in treatment-related adverse events and infections reported at 12 weeks compared with placebo at various doses. Thus, the overall treatment regimen of upadacitinib 15 mg once daily along with the csDMARDs was more likely to be the best treatment to achieve an ACR20 response and relatively low adverse events; this may be because each drug has a different mode of action when used in rheumatoid arthritis. DMARDs, such as Methotrexate, work by inhibiting lymphocyte activation and proliferation, whereas upadacitinib, a selective JAK1 inhibitor, improves treatment efficacy in rheumatoid arthritis [4,18].

A Bayesian network meta-analysis by Song GG et al. [19] also showed that upadacitinib 15 mg or 30 mg, along with Methotrexate, was the most effective treatment regimen compared with upadacitinib monotherapy. One of the reasons behind this might be how upadacitinib works by selectively inhibiting JAK 1 and Methotrexate by affecting the activation and proliferation of lymphocytes. Methotrexate, apart from inhibiting the enzyme dihydrofolate reductase, has also decreased viability and cellular proliferation, especially of T lymphocytes, which play an essential role in disease progression. Methotrexate, by inducing apoptosis by increasing oxidative stress, may have been linked to its beneficial role in rheumatoid arthritis patients. When used with upadacitinib, it has shown a synergistic relation in managing patients [18]. Many experts also concluded that upadacitinib (15 mg or 30 mg once daily) showed radiographic inhibition in rheumatoid arthritis and was superior to placebo in Methotrexate insufficient responders [20].

The number of patients getting treated with upadacitinib showed relatively more treatment-related side events; however, when compared among the individual doses that were approved for clinical use, 15 mg or 30 mg once daily, there was no difference from that in the placebo, except in the occurrence of any adverse event, infection, or any severe infection at 30 mg once daily, which occurs more with patients receiving upadacitinib. Upadacitinib has been notorious for showing a high proportion of side effects in some studies, such as an increase in total high-density lipoprotein and low-density lipoprotein cholesterol, most likely due to the mechanism of IL-6 pathway blockade and an increase in liver enzymes and also creatine kinase levels [9,10]. Although no significant difference was found in our analysis for any hepatic adverse event or herpes zoster infection, Nader et al. [21] found that a 15 mg once daily upadacitinib regimen provided an optimal benefit-risk profile. However, patients receiving upadacitinib should be monitored closely for any long-term adverse events, especially infection. 

Our study had some limitations; the follow-up time was limited to 12 weeks, which made us exclude a paper by Fleischmann E et al. [11], whose safety outcome was measured at 26 weeks, making the duration too short to evaluate the long-term effects. We only measured efficacy in patients who achieved an ACR20 response at 12 weeks without evaluating other outcomes. Nevertheless, this meta-analysis has several strengths based on 3233 patients. We provided more accurate data than individual studies by increasing the statistical power and independent analysis of the efficacy and safety of upadacitinib at the different doses tested in patients with active rheumatoid arthritis.

## Conclusions

In this meta-analysis, we involved the data of 3233 patients from six randomized controlled trials, which showed that 67.5% of patients responded well to upadacitinib compared to placebo (36.1%) in achieving ACR20 response. Upadacitinib, administered at the dose of 15 mg once daily in combination with Methotrexate, was the most efficacious treatment regimen option for patients with active rheumatoid arthritis compared to a 30 mg once daily dose. It was also associated with a significantly low risk for treatment-related adverse events, and maximum adverse events were reported at 12 mg twice daily.

## Appendices

**Table 2:** Cochrane risk of bias assessment for included studies. Source References: [7-12]

	Random sequence generation (selection bias)	Allocation concealment (selection bias)	Blinding participant & personal (performance bias)	Blinding outcome assessment (detection bias)	Incomplete outcome date (attrition bias)	Selective reporting (reporting bias)
Kremer et al. [7]	+	+	+	+	+	+
Genovese et al. [8]	+	+	+	+	+	+
Burmester et al. [9]	+	+	+	+	+	+
Genovese et al. [10]	+	+	+	+	+	+
Fleischmann et al. [11]	+	+	+	+	+	+
Kameda et al. [12]	+	+	+	+	+	+
